# Ophthalmology

**Published:** 1900-01

**Authors:** Alvin A. Hubbell

**Affiliations:** Buffalo, N. Y.


### Progress in Medical Science.

### OPHTHALMOLOGY.

Conducted by ALVIN A. HUBBELL, M. D., Buffalo, N. Y.

### SUPPRESSION OF THE CRYSTALLINE LENS IN MYOPIA.

PROF. PFLÜGER, of Berne, has an elaborate paper in the *Bulletins et Memoires*, of the French Ophthalmological Society, covering 197 octavo pages, which may truly be considered a classic on the subject of the removal of the crystalline lens in myopia. The burden of the paper is an analysis of 103 cases (108 eyes) operated upon by him between 1891 and 1896, of which 95 have been followed up. Incidentally, he reviews the history of this method of treating myopia, clinical ophthalmometry, optometric determination and mathematical expression of the degree of ametropia, and the diminution of refraction by aphakia. After detailing 101 cases, he follows with a consideration of their etiology, the influence of heredity, their ages at the time of operation, and the conditions, as to degree of myopia, age, acuteness of vision, and pathological appearances, indicating an operation. In regard to the operation itself and its results, the following abstract, taken from the *Ophthalmic Review*, October, 1899, will give the reader the gist of the author's experience :

It is in the cure of high myopia that removal of the transparent lens presents the greatest theoretical and practical interest. As regards the two serious objections to the operation, loss of accommodation and detachment of retina, the pernicious consequences of accommodation on the nutrition of the myopic eye are removed, whilst nothing justifies the fear of a predisposition to retinal detachment caused by the aphakia, the results pointing rather to a prophylactic influence. The operation should not be blamed for causing a detachment, unless it has been accompanied by complications. From the end of 1891 to the end of 1896 Pflüger operated on 103 cases (108 eyes) and has been able to follow up 95. He points out that it is necessary to be exact in the estimation of the ametropia; the astigmatism and the radius of curvature of the cornea being determined by Javal's ophthalmometer, the state of the refraction might be directly expressed by the neutralising lens placed at the anterior focus of the eye, according to the ophthalmometric method of Giraud-Teulon. Observations show that removal of the lens is not always followed by the same diminution of refraction in eyes which had the same degree of myopia. This is due to errors in the determination of the ametropia. Those errors due to spasm of accommodation can be avoided by the use of atropine, and those due to insufficient correction of astigmatism by the employment of the ophthalmometer. Errors, more or less inevitable, result from the difficulty of placing the correcting glass at the anterior focus of the eye, which varies according to the radius of curvature of the cornea. The errors resulting from position, degree of curvature, and index of refraction of lens or aqueous are negligible. According to a formula, established by Pflüger for the approximate prognostication of the refraction after aphakia in different degrees of myopia, a myopia of 16D., with a corneal refraction of 50D., will become emmetropia, as also myopia of 18D., with a corneal refraction of 45D., and a myopia of 20D. with a corneal refraction of 41D. or 42D.

Of the ninety-five cases which Pflüger analyzed, eighty-nine had one eye operated on, and six had both done. Hereditary influence was noted in 52 per cent. of cases; and in 91 per cent. the myopia was congenital. The operation was done in eighty-three cases between the ages of 7 and 30; in twelve cases between 30 and 40; in five between 40 and 50; and once at 51. Patients from 7 to 10 years with high myopia ought certainly to be operated on, and even if the myopia is below 12D. and there is a fair corneal curvature, we should not wait for irreparable changes in the fundus or vitreous before interfering; for a myopia of this degree is to be looked upon with suspicion, and operation gives a better guarantee for the maintenance of the status quo than wearing glasses.

For determining in what cases it is justifiable to interfere it is necessary to consider not only the degree of myopia, but also the age of the patient, the mean corneal refraction, the visual acuity, the general condition of the patient, his social position, and the progress of the myopia in himself and his family. If reduction in visual acuity compels the patient to change his profession, operation would be indicated for a myopia distinctly below 16D. Rapid increase in the myopia before 20, and particularly after that age, would be an indication for operating even in moderate degrees of myopia.

Pflüger's cases have been followed up for a period of from two to seven years and allow of interesting deductions. The cases are divided into three classes, according to the degree of myopia at the time of interference. The first class comprises myopes of 10D to 13D. Three cases, from 9 to 11 years, were operated on for a myopia of 10D.,with a resulting hypermetropia of from 3.75D. to 5.75D., and a marked increase in visual acuity. One case was attacked with choroiditis two years after operation, which seriously affected the vision. In five cases of myopia of 11D., three were aged from 8 to 12 years, one was aged 15, and one aged 20; the resulting hypermetropia was 2.5D. to 5D., and the visual acuity in all cases was improved. Of twenty cases of 12 to 13D., fourteen were below 20 years, and six between 20 and 28 years. The resulting hypermetropia varied from 2D. to 5D., and the vision was improved in all the patients but one, whose sight was lost from detachment of the retina ensuing some years after the operation.

The second class comprises myopes of 14D. to 15D. In twelve cases the myopia was 14D. and in four it was 15D., the age varying from 9 to 23 years in 14 patients, one patient being 30 and another 40. Four times there resulted emmetropia in one meridian, the other being corrected by concave cylinders of from 1D. to 2D. In the other cases the correcting spherical lenses varied between 1D. and 4D.

The third class includes fifty-three cases of myopia of 16D. to 29D. The age of the patients varied between 7 and 51 years. In one case of 16D., three cases of 20D., and one case of 21D., the refraction became emmetropic after operation. In nineteen cases the refraction was still myopic, one case having an initial myopia of 17D.,three cases of 18D. and the others of 19 to 20D. The myopia or hypermetropia resulting in all cases never exceeded 4d.

Visual acuity was improved in all cases but two; in one case there was a secondary infection following the discission, and in the other a central retinal hemorrhage in the course of an attack of delirium tremens. In addition two eyes have lost their vision after some years, one from retinal detachment and the other from severe choroiditis.

Pflüger thinks that the improved visual acuity depends on a better nutrition of the organ leading to an improvement in the function of the retina, as well as on the development of the function of the occipital lobe by distant vision. He has in most found an improvement in the visual field and color sense. In his own cases he has not found one in which the myopia made any disquieting progress during the period of from two to six years after operation. In most unfavorable cases the elongation of the antero-posterior axis was less in the eye operated on than in its fellow.

The comparison of the two eyes leaves no doubt of the prophylatic action of the operation. Pflüger considers extensive corneal opacity as about the only real contraindication. Neither myopic choroiditis nor old central choroiditis with definite loss of central vision contraindicate. Severe acute central choroiditis is, moreover, an urgent indication for operation. Discission not extending to the posterior capsule and followed by simple extraction aided by the curette is recommended in all cases.

Increase of tension, even if painless, should call for linear extraction. The lance should be inserted in the limbus and the curette be used only to evacuate loose masses of lens substance, the operation being reduced in a manner to simple paracentesis, which can be repeated several times without danger. With regard to flap extraction, it should be reserved for those ages when a hard nucleus is expected or in cases where a first discission has proved the existence of such a nucleus. In the discission of secondary cataract Pflüger lays stress upon the danger of interference with the vitreous and prefers using the "pince-ciseaux" of De Wecker, or the harpoons of Stelling. With regard to post-operative complications, infection of the wound is possible in spite of all precautions, and detachment of retina, Pflüger holds, is merely a question of chance; for although loss of vitreous should be avoided, it is not only the factor in producing detachment, since in the one case in which it ensued there had been no loss of vitreous, and nine cases in which vitreous had been lost did not become affected with detachment. Glaucoma has not resulted, but it could be met by iridectomy. It is difficult to say whether vitreous opacities may be the consequence of intervention.

Prof. Pflüger concludes his valuable paper with an extended bibliography.

